# Prevalence of Breast Cancer in a Defined Population of Iran

**DOI:** 10.5812/kowsar.20741804.2245

**Published:** 2011-09-15

**Authors:** A Rezaianzadeh, S T Heydari, H Hosseini, A A Haghdoost, E Barooti, K B Lankarani

**Affiliations:** 1 Department of Epidemiology, Shiraz University of Medical Sciences, Shiraz, Iran; 2 Health Policy Re-search Center, Shiraz University of Medical Sciences, Shiraz, Iran; 3 Kerman University of Medical Sciences, Kerman, Iran; 4 Ministry of Health and Medical Education,Women’s Affairs Office, Tehran, Iran

**Keywords:** Prevalence, Breast cancer, Household women, Iran

## Abstract

**Background:**

Prevalence of breast cancer in Asian developing countries is much lower than western developed countries. The main aim of this study was to measure breast cancer prevalence in a defined population of Iran.

**Methods:**

A total of 25201 women who were under coverage of “Imam Khomeini Relief Foundation (IKRF)”, which is an organization for delivering supportive social and cultural services to the deprived and poor subgroups of the society, were involved in the study. The study was conducted during years 2007 and 2008. All subjects were interviewed for their socio-demographic features and underwent precise clinical and para-clinical breast examination.

**Results:**

Mean age was 47 years with standard deviation 10 ranging from 11 to 88 years. Subjects were from deprived subgroups of the community; were mainly illiterate or had primary school education (86%) and majority of them (93%) had their first full-term pregnancy at age less than 26 years and also were multiparous. With confirmed diagnosis by breast biopsy, breast cancer prevalence was 0.15% (95%CI; 0.10-0.20).

**Conclusion:**

Compared with developed countries, Asian developing countries have been at a lower risk of breast cancer development. It is seen that more deprived subgroups are at much lower risk. The more industrialized life is accompanied with more hazards.

## Introduction

Breast cancer is the most frequently diagnosed malignancy among women in developed countries,[1][2][3] and in some developing countries.[4][5][6] Breast cancer is more prevalent in developed western countries than in developing Asian countries. Investigations have revealed that almost one in nine American women will suffer from breast cancer in their lifetimes[7][8] while the Western European and North American population have the highest lifetime risk. Prevalence of breast cancer in these countries is estimated between 8 and 10%. However, the lowest prevalence is seen in Asian countries, about 1%.[9] In Iran, the prevalence of breast cancer was reported to be 6.7 per thousand in 2002, which is less than the esti-mates for Asian countries.[10] Several reports from Iran have reported that the prevalence of breast cancer in Iran is lower than in European and American countries but they did not report an exact measure.[11][12]

Breast cancer usually occurs at the time when women have important family and occupational roles, and thus any treatment in relation to this cancer would be particularly stressful for the patient and her family. Disparities in the prevalence of breast cancer have long been debated. As mentioned before the prevalence of breast cancer is low in Iran. The main aim of this study was to measure the prevalence of breast cancer in a specific group of women from low social class and deprived subgroups of Iranian population.

## Materials and Methods

A total of 25201 women who were insured by the “Imam Khomeini Relief Foundation (IKRF)” were involved in this study. The “Imam Khomeini Relief Foundation (IKRF)” was established in 1980 in Islamic Republic of Iran for delivering supportive

social and cultural services to deprived and poor subgroups of the society. Thus women in this study were mainly Vulnerable Household Women’s Health Assessment (VH-WHAT) from poor and low social subgroups1.[13] This study was conducted during a two years period from 2007 to 2009.

Islamic Republic of Iran, in the Middle East region with an over 70 million population, consists of 30 provinces with Tehran as its capital with more than 10 million residents. Along with Tehran, 10 more province capitals including: Shiraz, Mashhad, Kerman, Kermanshah, Bushehr, Qom, Isfahan, Gorgan, Rasht and Yazd were included in this study.

The statistics and number of vulnerable women was obtained from the provincial public service organizations (IKRF). Population size of each province capital was determined according to its population and allocated budget.

All of the subjects were first interviewed for socio-demographic data, drug history and history of cancer. Selected gynecologists and surgeons attended educational workshops to be oriented regarding the principals of a concise breast examination. Then in the appointment day, a gynecologist or surgeon performed a complete breast examination to detect any lump or mass along with axillary lymph node exam. All par-ticipants in the range of 35 to 60 years then referred to designated radiology centers for mammography. For those women above 60 or below 35 years of age, mammography was done according to physician’s opinion and results of breast examination.

In some cases with suspicious results of mammography, a breast ultrasonography was performed to evaluate any abnormal mass or lumps; in presence of any cystic lesion it was aspirated by needle. All suspicious breast masses underwent biopsy for detection of malignant lesions and the breast cancer diagnosis was confirmed by the result of biopsy. All analysis was conducted using Stata v10.

## Results

Mean age of the population study was 47 years with standard deviation of 10 years, ranging from 11 to 88 years. Only 2% were single while 19% were married and 79% were divorced or widowed women. Fifty eight percent reported their menarche at age 13 years or less and the rest at 14 years and above. Age of marriage in 70% of them was 18 years or less and in 26% was 19 to 25 years and only 4% had age of marriage at age 26 years or above. Fifty five percent had their first pregnancy at age 18 or less and 37% between age 19 to 25 and only 8% at age 26 years and above. Eighty eight percent were unemployed and received their life expenses from IKRF. Forty percent were illiterate and 47% had only primary school education and only 3% had university education. Forty six percent had never used OCP, 47% had started to use OCP between ages 20 to 30 years and 7% at age above 30 years. However, duration of OCP use was not clear for the two last groups (Table 1).

**Table 1:** Distribution of socio-demographic and reproductive features

**Variable**	**Percent**	**Variable**	**Percent**
Marital status		Education	
Single	2	Illiterate	39
Married	19	Primary school	47
Divorced	29	High school	11
Widowed	50	University	3
Occupation		Menarche age	
Manual	6	≤13 years	58
Non-manual	5	≥14 years	42
Housewife	89		
Age at marriage		Age at first pregnancy	
≤18 years	70	≤18 years	55
19-25 years	26	19-25 years	38
≥ 26 years	4	≥26 years	7
No. of pregnancies		OCP use	
0	5	Never	46
1-5	64	Between age 20-30	47
6-10	27	Above age 30	7
≥11	4		
Age at time of study		Smoking	
≥ 25 years	1	Smoker	3
26-39 years	23	Ex-smoker	2
40-49 years	37	Never smoker	95
50-64 years	36		
≥ 65 years	3		

Number of pregnancies was significantly associated with diagnosis of breast cancer and those with a higher number of pregnancies were less likely to be diagnosed with breast cancer. Marital status, menarche age, age of marriage, age at first pregnancy, occupation and education were not associated with the disease diagnosis.

About 1% of subjects had history of radiation exposure and 6% had reported hormone therapy before the time study and these two factors were not associated with the disease diagnosis. Positive family history of both breast cancer and ovarian cancer was about 3%. Also family history of breast and ovarian cancer were significantly related and those with family history of one cancer were more likely to have family history of another. Eighteen percent of breast cancer patients had reported family history of all other cancers. Only one of the breast cancer patients had been previously diagnosed with ovarian cancer. History of cancer in subjects and their families was not associated with the disease diagnosis.

All of the subjects were examined for benign changes in breast skin and nipple like redness and exfoliation. However, none of diagnosed patients with breast cancer had these changes in their breasts. Final diagnosis of breast cancer was confirmed in 38 of 25201 subjects of study which was 0.15%, 95%CI; 0.10-0.20.

## Discussion 

The results of this study showed that prevalence of breast cancer in the specific population of study was 0.15% which is much lower than other studies on breast cancer prevalence from Iran.[10][11] The majorities of subjects in this study (93%) had their first full-term pregnancy at age less than 26 years and also were multiparous. Furthermore, those with a higher number of pregnancies were less likely to be diagnosed with breast cancer. It has been shown that early pregnancy and multiparity are protective factors for breast cancer development.[13] About half of subjects of this study had never used OCP and the rest had reported OCP use between ages 20 to 30 years without mentioning the duration of usage. In addition, apart from use of OCP, 94% of subjects had reported no hormone replacement therapy. It has been also reported that prolonged exposure to exogenous estrogens and progestins in hormone therapy increased a woman's risk of developing breast cancer.[14]

Eighty six percent of our study population were illiterate or had education level less than 6 years. A 36% lower risk was observed for women with more than 16 years of education as compared to those with the lowest educational achievement (7-9 years).[15] Alcohol intake was another risk factor for breast cancer and none of subjects of this study were alcoholic.[15]

These characteristics of our study population could be a reason for the very low prevalence of breast cancer among them. Moreover, all subjects were from a low social class and deprived subgroups of Iran's population. Although recent studies have shown that breast cancer risk is increasing in developed countries, some studies reported that changes in risk of breast cancer differs in various thnicities.[15][16] The prevalence of breast cancer is much higher in developed countries but with a better prognosis. In Iran with a lower prevalence, it has been shown that the overall prognosis is worse when compared to de-veloped countries.[17][18]

Furthermore, despite the high prevalence, the prognosis of breast cancer is better in the developed countries. However, although the prevalence of breast cancer in Iran is lower than that in the developed countries, the overall prognosis is worse.[19] Since this study was conducted on a specific group of Iran's population, further investigations on general population are needed to measure the prevalence of breast cancer in normal population.
